# Structure in transition: The role of structure in facilitating workplace efficacy and belonging for military veterans and civilians

**DOI:** 10.1371/journal.pone.0317575

**Published:** 2025-02-03

**Authors:** W. Connor Gibbs, Lauren Ortosky, Alair MacLean, Aaron C. Kay, David K. Sherman

**Affiliations:** 1 Department of Psychological and Brain Sciences, University of California, Santa Barbara, Santa Barbara, California, United States of America; 2 Department of Sociology, Washington State University, Pullman, Washington, United States of America; 3 Fuqua School of Business, Duke University, Durham, North Carolina, United States of America; 4 Department of Psychology and Neuroscience, Duke University, Durham, North Carolina, United States of America; Dayeh University, TAIWAN

## Abstract

Employment transitions necessitate a degree of uncertainty and lack of control, which may present a challenge to succeeding and belonging at a new organization. The present research tests ideas derived from compensatory control theory which posits that people may seek external structure to help exert control over their lives when they experience a lack of control in an important life domain – and that this can aid in their goal pursuit. Across three studies, we explore whether the perception of a higher degree of organizational structure can help employees compensate for uncertainty and lack of control and facilitate transitioning employees’ occupation self-efficacy and sense of belonging in a new work environment. This research focuses on military veterans, who face significant challenges during their separation from military service and transition to civilian employment, as an exemplar of the people experiencing employment transitions more generally, and compares them (in two studies) with civilian participants. Across three studies, two using simple correlational methods, one using an experimental methodology with veterans and civilians, we find consistent evidence that when transitioning employees perceive greater structure at their organization, this facilitates increased feelings of occupational self-efficacy which, in turn, promotes greater feelings of belonging at work. When people perceive greater structure in their environment, people feel more efficacious and a greater sense that they belong at work. The results are discussed in the context of compensatory control theory, addressing the challenges of transitioning employees, and in particular, transitioning military veterans.

## Introduction

Major life transitions challenge people’s ability to predict and control their environments. However, the beliefs and identities that individuals hold as they work through these transitions can impact their motivations and transition outcomes [1,2]. Transitioning from the military to civilian life and the workforce is an acute example where loss of environmental structure and routine can exacerbate employment transition challenges. Yet, the success of a transition is also a function of what situation people are transitioning into. When an organization provides a significant degree of structure, people may be less likely to feel lost and more likely to feel as though they can succeed and achieve their goals. Adding to a growing body of social psychological research on military veterans’ civilian employment experience [3], in the present work, we sought to examine the relationship between the amount of structure people perceive during their employment transitions and their transition outcomes – the extent to which they feel as though they can succeed and belong in the organization, with a particular focus on transitioning employees who have served in the military.

### Transitions as a threat to personal control

Employment transitions, such as when people begin their first occupation after completing their education or military service, can act as an external threat to individuals’ experiences of personal control. For example, during their transition to civilian employment, military veterans have reported experiencing anxiety as a result of their civilian employers’ lack of a clearly defined onboarding process [4]. More generally, employment transitions require navigating opaque hiring processes (e.g., application review, interviews, assessment testing, hiring decisions) that candidates often have little control over. Likewise, once an individual finds a new place of employment, they frequently lack control over their work environment, job responsibilities, and expectations. When college graduates transition to the workforce, they may feel pressure to adapt their identities, responsibilities, relationships, lifestyles, and level of independence to their new employment status [5], setting an expectation of adversity for many graduates [6]. With major life transitions come a series of challenges, and transitioning into the workforce is a prime example [7].

When people have experiences that are stressful, chaotic, and unpredictable, such as may occur during employment transition, they engage in a variety of coping strategies. Research and theorizing on compensatory control has focused on how people strive to perceive the world as orderly, where all events follow clear cause and effect relationships, and how these perceptions may aid them in their ability to cope with an otherwise chaotic perception of the world [8]. Wanting to perceive the world as non-random and orderly is argued to be a fundamental human motive [9–12]. Compensatory control theory (CCT) [8,13] argues that this perception that the world is structured and orderly facilitates the development of feelings of personal control – defined as “an individual’s belief that [they] can personally predict, affect, and steer events in the present and future” [13], p. 264. CCT goes on to argue that when individuals perceive a lack of personal control, for example following an external threat to their control such as employment transition, they engage in psychological processes to reinforce their foundational perception that the world is orderly, rebuilding the foundation upon which personal control and individual goal pursuit may be developed.

In this paper, we explore the implications of this theorizing for the experience of employment transition, with a particular focus on those who are making the transition from the military to their first post-military work experience. We examine whether people respond to the potential lack of personal control they experience during an employment transition by drawing on their perceptions of the new workplace, and in particular, being attuned to and impacted by the amount of structure they perceive as a potential strategy to compensate for a relative lack of control they may be personally experiencing [8,14]. The experience of structure in initial employment varies widely for people as a function of where they work, how the workplace is constructed, and their perceptions of that structure. In this research, we seek to better understand the implications of this variation on the perceived success of individual employment transitions, as it may have implications for how best to structure environments to promote transitional success.

### Military veterans as an exemplar group for research on transitions

Although it may be the case that sources of structure provided by an employer are impactful to all transitioning employees by potentially facilitating their goals, in this research we explore whether structured employment environments are particularly beneficial to those who have been socialized to expect a structured work environment (e.g., military veterans). The military is a “total institution,” that requires its members to fully embrace “the values, norms, and practices” of the military [15,16]. Through the total institution of the military, service members are exposed to a near continuous reminder that their world is regimented, structured, and hierarchical with specific expectations and norms regarding interpersonal conduct, discipline, and obedience [16–20]. Military service provides structure through daily routine and a clear hierarchy of command, including the clarifying and simplifying of tasks that generate a system of opportunities to excel and a regular pathway of advancement [17,21,22]. Becoming socialized to expect such order and structure from one’s environment may make military veterans particularly reliant on structure in their environment to facilitate the pursuit of their goals after being discharged, knowing that civilian organizations tend to be flexible and egalitarian [23], rarely providing similar structure to that provided by the military [24].

Fostering success among transitioning military veterans has long been a goal of both private industry and the government [25], and yet, there continues to be a wide range of challenges facing transitioning veterans [3]. According to the United States Department of Labor, 200,000 military veterans transition to civilian life every year [26]. United States (U.S.) military veterans report that beginning a civilian career can be challenging. In a sample of 2,044 veterans, 40% describe their transition to civilian employment as “difficult” or “very difficult” [27]. Likewise, research based on analyses of veteran and civilian LinkedIn users reveals that underemployment, defined as working an hourly wage job while having a bachelor’s degree or higher, is a significant and increasing issue for veterans [28]. Whereas in 2010 veteran underemployment was at roughly 11% and civilian underemployment was at about 12%, in 2019, civilian underemployment remained at about 12%, while veteran underemployment rose sharply to roughly 34% [28].

Military veterans beginning their civilian careers face numerous, complex challenges including transitioning from a military culture to a civilian culture [29], adapting to new social dynamics [3], and maintaining psychological health after potential trauma experiences [30,31]. Each of these factors represent significant challenges to veterans as they pursue their goals in their new, civilian employment. Adding to these challenges is the stark contrast between the extent of structure found within military service relative to civilian life. In contrast to military life, civilian life can be far less structured as a result of its variety and lack of routine. Upon discharge and a return to civilian culture, veterans’ environments are likely to become significantly less structured, exacerbating the loss of structure and control veterans face during transition and presenting difficulties adapting to their new reality [3]. Not only must veterans cope with a loss of personal control inherent to transitions themselves, but they must also adapt to a new environment with *less* structure, order, and predictability than that to which they had been accustomed. And while in aggregate it may be the case that military service is more structured than civilian life, the significant heterogeneity among service members’ military experiences as a function of their branch and era of service, service role, and if a veteran joined the reserves immediately after service, may make the structure in military service and civilian life more or less similar for individual veterans.

We propose that how military veterans subjectively construe the loss of environmental structure that may mark the start of their civilian lives is a key psychological driver of some of the occupational challenges they face when transitioning [32]. This loss of structure may make it challenging for veterans to shore up their foundational belief in an orderly and predictable world in such a way that enables them to pursue their goals [14]. In the present research we thus sought to explore the relationship between perceived structure in a new employment environment and key transition outcomes among military veterans and civilians (i.e., non-veterans).

### Structure as control compensation

Our central premise is that because employment transitions represent periods of low personal control, transitioning employees may look for sources of structure within their new work environments as a means of building (or rebuilding) that sense of control. In the present work, we define workplace structure as any element of one’s employment that provides or imposes some degree of order or predictability, including elements such as corporate rules and expectations, work routines, and hierarchical leadership. Past research has shown that organizational structure can impact processes of information processing, decision making, and employee performance [33–37]. Sources of structure may help transitioning employees compensate for feelings of low personal control and maintain their perception that the world is orderly and predictable. Past research has shown that sources of organizational support, such as alternative scheduling and support from one’s supervisor and coworkers, is associated with greater perceived control [38], highlighting how systems within one’s employment environment may facilitate the development of personal control. By helping to satisfy one’s core motivation to perceive the world as orderly, non-random, and potentially controllable [39], perceiving greater structure in one’s new work environment may lead to a more successful employment transition. By contrast, if new employees transitioning from a different career subjectively construe their new work environment as lacking structure, predictability, and order, the transition may be particularly difficult.

Such responses would be consistent with compensatory control theory, which explains how and why, during periods of low personal control, individuals come to rely on control compensation strategies to help them strengthen their perceptions of an orderly and predictable world. For example, individuals in states of low personal control turn to external agents they see as benevolent as sources of control and structure in their life, such as God or the government [8,13,39,40], and this helps them to maintain a global perspective that the world is orderly and predictable, which can then enable effective goal pursuit. Indeed, simply being exposed to world events that are seen as highly structured (e.g., the earth’s orbit around the sun, the tides of the oceans, and variations in traffic congestion throughout the day) can lead to increased motivation and action towards one’s goals [41]. When one feels a lack of control, being reminded that the world follows a consistent pattern with clear cause and effect helps individuals to see that their efforts toward their goals are not in vain. This could help to rebuild their feelings of control and motivation to pursue their goals. In the employment context, then, we examine whether pursuing one’s goals in the workplace – to do well and to fit in at work - is facilitated when people see their employment as relatively structured, compared to relatively lacking in structure.

### Occupational self-efficacy and belonging in the workplace

In the present work, we focus on two key outcomes that are relevant to pursuing employment related goals: occupational self-efficacy and sense of belonging in the workplace. We reason that if individuals struggle to perceive structure during potentially control-threatening career transitions, then it may be particularly challenging for them to effectively perform in their jobs and feel as though they belong in the workplace.

Occupational self-efficacy “refers to the competence that a person feels concerning the ability to successfully fulfill the tasks involved in [their] job” [42], p. 239. Occupational self-efficacy has been shown to promote both work performance and intrinsic motivation [43], as well as commitment to one’s organization and work engagement [44]. Prior research has demonstrated that providing external structure to individuals can improve their self-efficacy related to pursuing goals [45]. Individuals who perceived their work environment as having greater procedural justice and being more hierarchical reported a greater sense of self-efficacy in their work [45]. We theorize that transitioning to an organization that one perceives as more structured will be positively associated with occupational self-efficacy whereas transitioning to an organization that one perceives as less structured would lead people to feel less efficacy in the workplace. To the extent that people are able to draw upon their subjective construal of structure in their work environments (e.g., more clear hierarchy, fixed routines, clearer expectations), it will, we predict, facilitate their pursuit of occupational goals, including successfully executing in their work performance, thus exhibiting occupational self-efficacy.

We further reasoned that greater organizational structure could lead to a greater sense of belonging at that organization. One of the basic human motivations is the need to belong [46,47], and in organizational contexts, this may manifest itself as feelings of being a respected and esteemed member of the workforce and connected to one’s coworkers and the larger organization [48]. This feeling of belonging in the workplace might be augmented to the extent one believes oneself to be performing efficaciously at work. In employment settings, individuals are often initially evaluated by their ability to perform their job well, incentivizing the prioritization of being an efficacious and successful employee. Current employees may hesitate to socially connect with and may even punish an employee who performs poorly at their job [49]. Likewise, while poor job performance may elicit negative feelings from one’s manager and coworkers, contributing to a perceived lack of belonging, strong job performance can lead to more favorable views of employees by managers [50]. Moreover, inadequate job performance, by appearing to confirm negative stereotypes and triggering stereotype threat, has been shown to have detrimental impacts on underrepresented employee’s and students’ sense of belonging [51].

To sum up, we predict that a downstream consequence of increased organizational structure, because of its effects on occupational self-efficacy, will be increased feelings of workplace belonging.

### Overview of studies

Across three studies, we test how perceived organizational structure during times of employment transition may impact, or be associated with, occupational self-efficacy and sense of belonging. Study 1, using a sample of military veterans and civilians (i.e., non-veteran former students with some level of formal education) who reflected on their transitions to their first jobs, tested whether greater perceived organizational structure was associated with increased occupational efficacy and belonging at work. We also examine, in a study reported in supplemental materials, whether heterogeneity in the veteran experience on branch and era of service, service role, and if a veteran joined the reserves immediately after service, moderates the impact of perceived structure on these outcomes. Study 2 (preregistered) tests the robustness and generalizability of the previous findings by examining the proposed relationship at veterans’ current employers, as opposed to previous employers, using narrower single item measure of our key variables. Studies 1 and 2 adopt a mediational approach to examine whether efficacy mediates the relationship between structure and belonging. Finally, Study 3 (preregistered) adopts an experimental methodology to examine whether manipulating perceived structure leads to changes in anticipated efficacy and belonging.

### Overview of research samples, analytics, and methodology transparency

Tables 1 and 2 provide demographic information for all studies. Throughout the analyses reported in this paper, we will utilize control variables, notably age, gender, and race (see results sections for relevant descriptions of how these variables are coded). As indicated in Table 1, the veteran and civilian samples in both Studies 1 and 3 differ on these demographic characteristics. As such, in Study 1, these covariates will be used to help account for between group differences between veterans and civilians. For Study 3, due to its experimental designs, we will report findings from analyses without covariates included, and note any changes in results caused by the inclusion of covariates. Additionally, in Studies 1 and 2 (correlational), covariates will be used to help isolate effects of the psychological predictor on the outcomes above and beyond the impact of the individual differences (e.g., age, gender, race) captured by the covariates.

**Table 1:** Personal demographics.

		Study 1		Study 2	Study 3	
		Veterans (*N* = 149)	Civilians (*N* = 101)	Veterans (*N* = 497)	Veterans (*N* = 200)	Civilians (*N* = 200)
Gender	Male	62.4%	71.0%	80.5%	76.0%	34.0%^3^
	Female	37.6%	29.0%	15.7%	22.5%	64.0%
	Transgender Male	–	–	–	0.5%	0.5%
	Nonbinary/Gender Non-conforming	–	–	1.21%	1.0%	1.5%
	Prefer not to answer/Did not respond	–	–	2.61%	–	–
Age [*M* (*SD*)]		33.7 (8.07)	33.1 (7.75)	N/A^1^	42.9 (11.3)	38.5 (11.6)
Ethnicity	African American/Black	10.7%	9.0%	11.9%	4.5%	4.5%
	Asian American/Asian	4.03%	4.0%	7.04%	1.5%	5.0%
	European American/White	72.5%	75.0%	56.9%	85.5%	82.0%
	Hispanic American/Latino	6.04%	9.0%	–	2.5%	1.5%
	Native American or Pacific Islander	4.03%	3.0%	8.11%	1.0%	–
	Multi-Racial	2.68%	–	12.3%	2.5%	2.0%
	Other	–	–	8.65%	2.5%	5.0%

The demographics of the veteran and civilian samples for each study. In Studies 1 and 2 all participants were from the U.S. In Study 3, participants were recruited predominantly from the U.S. and United Kingdom (U.K.). The veteran sample of Study 3 was 58.0% from the U.S., 40.5% from the U.K., and 0.5% from another country. The civilian sample of Study 3 was 90.0% from the U.K., 3.0% from the U.S., 7.0% from another country).^2^

^1^Age data was not collected continuously. Median age range for Study 2 was “41–45 years old”.

^2^This disparity in the proportion of each sample that is from the U.S. and U.K. is due to the time in which data collection began and the available sample of participants on Prolific. There is a large number of U.S. and U.K. civilians on Prolific, this resulted in participation slots for the civilian sample filling up quickly. Because data collection began in the late evening Pacific Standard Time (early morning Greenwich Mean Time), U.K. civilians were able to claim a majority of participation slots before U.S. civilians. On the other hand, there were relatively few U.S. and U.K. veterans on Prolific that were eligible for this study (i.e., had not participated in any previous studies). As such, participation slots for the veteran sample filled up less quickly, which allowed for more U.S. veterans to participate.

^3^While there is a significant gender disparity between the two samples in Study 3, this is unlikely to be a major confound as previous investigations have shown that need for structure does not systematically differ between genders [52,53]. Additionally, gender and the condition by gender interaction will be controlled for in Study 3 analyses.

**Table 2:** Military demographics.

		Study 1	Study 2	Study 3
Branch of service	Air Force	16.8%	12.3%	22.7%
	Army	51.7%	26.4%	42.8%
	Coast Guard	1.3%	1.2%	0.5%
	Marine Corps	8.7%	15.1%	6.7%
	National Guard or Reserves	6.7%	5.8%	1.0%
	Navy	14.8%	17.3%	25.8%
	More than one branch	–	21.9%	0.5%
Years in service [*M* (*SD*)]		N/A^1^	–	8.6 (7.0)
Years since discharge [*M* (*SD*)]		N/A^2^	11.3 (8.44)	12.8 (11.0)

The military service demographics of the veteran samples for each study.

^1^Years in service data was not collected continuously. Median years in service range for Study 1 was “5–9 years”.

^2^Years since discharge data was not collected continuously. Median years since discharge for Study 1 was “4 years”.

We describe our sampling plan, all data exclusions (if any), all manipulations, and all measures in the reported studies in this article on the Open Science Framework (OSF; https://osf.io/fc627/). Data, analysis code, and research materials for all studies, along with supplemental materials, are available on OSF. Data were analyzed using R Statistical Software (v4.3.1) [54] and the package boot (v1.3.28.1) [55,56], DescTools (v0.99.49) [57], dplyr (v1.1.2) [58], ez (v4.4.0) [59], ggplot2 (v3.4.2) [60], lm.beta (v1.7.2) [61], psych (v2.3.6) [62], pwr (v1.3.0) [63], reshape2 (v1.4.4) [64], and reghelper (v1.1.1) [65]. Study designs, hypotheses, and analyses for Studies 2 and 3 were preregistered (preregistrations available on OSF).

## Study 1

The aim of Study 1 was to examine both veterans’ and civilians’ retrospective assessments of the start of their civilian careers using a quasi-experimental design. We predict that perceiving greater organizational structure will be associated with positive transition outcomes for both veterans and civilians. We additionally predicted that having been socialized to expect more structure in their environment, organizational structure would be more strongly associated with beneficial outcomes (efficacy and belonging) for veterans compared to civilians. Finally, to the extent that one’s belonging in the workplace during transition is predicated on one’s job performance, we predicted that occupational self-efficacy would mediate the relationship between perceived organizational structure and sense of belonging.

After graduation, former students face a significant transition out of the education system and into the workforce [6]. Similar to veterans’ start to civilian employment, former students beginning their careers confront a threat to their feeling of control as they can face what may be an opaque hiring process, a new work environment, job responsibilities, and expectations. Despite the transition from education to employment lacking a dramatic change in environmental structure, as may be experienced by veterans leaving the military, because of the threats to control present during civilians’ transition to employment, environmental structure may also be beneficial in promoting occupational self-efficacy and belonging among non-veteran civilians. We test this in Study 1 by recruiting a sample of both veterans and non-veteran former students. Moreover, sampling both veterans and civilians who have completed at least some amount of college allows us to also explore possible between-group variability in the magnitude of the predicted relationships between the two groups.

### Methods

All studies received Institutional Review Board approval from the Human Subjects Committee of the University of California, Santa Barbara. All studies obtained informed written consent from participants prior to data collection.

#### Participants.

A sample of 149 U.S. military veterans and 101 civilians were recruited using Amazon Mechanical Turk (see Table 1 for sample demographics). Data collection began on January 19th, 2019, and concluded on February 6th, 2019. Our target sample size was at least 100 veterans and 100 civilians as this would have provided 80% power for detecting an effect (*r*) as small as 0.20. No data analyses were conducted prior to the completion of data collection. Our final sample size provided 80% power for detecting an effect (*r*) as small as 0.18. All participants were compensated $1.00 for their participation.

#### Measures.

Consenting participants completed an online survey examining “factors that may influence how people view different job opportunities.” To focus on a particular place of employment, all participants were asked to think about the first place that they were employed (after the military or college). Specifically, veteran participants were asked about their “experience in the very first organization where [they] worked after the military.” Civilian participants were asked about their “experience in the very first organization where [they] worked after completing [their] education.” For all studies, measures and materials without references were developed by the authors.

All participants’ perceptions of structure at their first civilian or post-education organization were measured using five items [45]. The five items were “The rules in this organization were clear,” “This organization provided a clear and structured mode of life,” “This organization provided a consistent routine,” “This organization provided a well-ordered life with regular hours,” and “There was a very clear hierarchy in this organization.” All items were measured on seven-point scales ranging from 1 (*strongly disagree*) to 7 (*strongly agree*). The scores of the five items were averaged to generate a composite, *M* =  5.23, *SD* =  1.11, α =  0.85.

Participants’ sense of efficacy at their civilian or post-education workplace was measured using a six-item scale adapted from the short form Occupational Self-Efficacy Scale [42]. The six items were “When I was confronted with a problem in my job, I was usually able to find several solutions,” “Whatever came my way in my job, I felt that I could usually handle it,” “My past experiences prepared me well for my occupational future at that job,” “I could remain calm when facing difficulties in my job because I could rely on my abilities,” “I met the goals that I set for myself at that job,” and “I felt prepared for most of the demands in my job.” All items were measured on seven-point scales ranging from 1 (*strongly disagree*) to 7 (*strongly agree*). The scores of the six items were averaged to generate a composite, *M* =  5.38, *SD* =  1.04, α =  0.90.

Participants’ sense of belonging at their first civilian organization was measured using a three-item scale adapted from the Sense of Social and Academic Fit scale [66]. The three items were “I felt like I belonged at that organization,” “I fit in well at that organization,” and “I felt comfortable at that organization.” All three items were measured on seven-point scales ranging from 1 (*strongly disagree*) to 7 (*strongly agree*). The scores of the three items were averaged to generate a composite, *M* =  4.99, *SD* =  1.35, α =  0.89.

### Results

#### Effect of veteran status on perceived structure, self-efficacy, and belonging.

We first examined whether veterans and civilians differ in the degree of reported perceived organizational structure, and self-efficacy and belonging at their first employer. Veterans (*M* =  5.24, *SD* =  1.15) and civilians (*M* =  5.23, *SD* =  1.04) reported equivalent levels of perceived structure, *F*(1, 243) =  0.01, *p* =  0.92, *η*^2^ =  0.0003. Likewise, veterans (*M* =  5.46, *SD* =  1.07) and civilians (*M* =  5.26, *SD* =  0.99) reported equivalent levels of occupational self-efficacy, *F*(1, 244) =  1.60, *p* =  0.21, *η*^2^ =  0.005. Finally, veterans (*M* =  5.05, *SD* =  1.38) and civilians (*M* =  4.90, *SD* =  1.30) also reported equivalent levels of belonging, *F*(1, 244) =  0.45, *p* =  0.50, *η*^2^ =  0.001.

#### Perceived structure predicts occupational self-efficacy.

**Fig 1:**
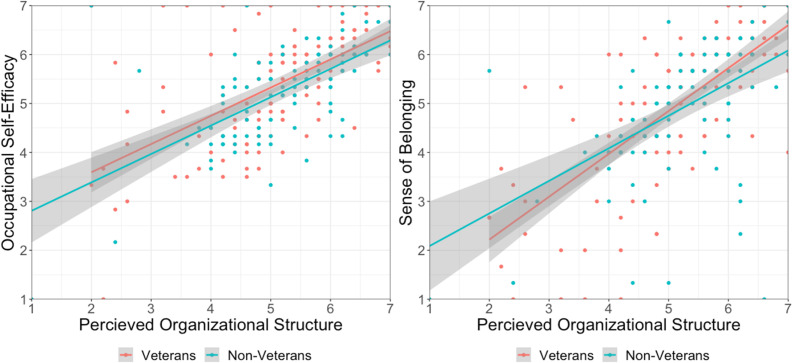
Perceived structure predicting efficacy (a) and belonging (b). Perceived organizational structure predicting occupational self-efficacy (*b*_veterans_ =  0.57, *b*_civilians_ =  0.53) and sense of belonging (*b*_veterans_ =  0.87, *b*_civilians_ =  0.60).

We next examined whether participants’ sense of how structured the workplace was at their first place of employment after transitioning was associated with increased workplace efficacy, and whether that varied by veteran status. We conducted a hierarchical linear regression to examine whether veteran status moderated the association between perceived organizational structure and occupational self-efficacy. We entered veteran status (1 =  veterans and 0 =  civilians) and mean-centered perceived organizational structure as predictors at Step 1 and their interaction as an additional predictor at Step 2. Age, gender (0 =  male, 1 =  non-male), and race (0 =  European American/White, 1 =  non-European American/White) were also included as covariates in both steps. Race was coded dichotomously to control for whether the participant was in the modal racial category within the sample. Unless otherwise noted, the same covariates were included in all additional analyses across all studies. Occupational self-efficacy was entered as the outcome variable. From Step 1, there was a significant main effect of perceived organizational structure, *β* =  0.59, *b* =  0.56, *SE* =  0.05, *t*(242) =  11.60, *p* <  0.001, 95% CI for *b* =  [0.46, 0.65]. Participants who perceived greater structure at their first civilian organization reported feeling greater self-efficacy in their work. The main effect of veteran status was not significant, *β* =  -0.08, *b* =  -0.18, *SE* =  0.11, *t*(242) =  1.64, *p* =  0.10, 95% CI for *b* =  [-0.39, 0.03]. From Step 2, the main effect of perceived organizational structure was not qualified by a significant interaction between perceived organizational structure and veteran status, *β* =  -0.03, *b* =  -0.04, *SE* =  0.10, *t*(241) =  0.40, *p* =  0.69, 95% CI for *b* =  [-0.24, 0.16]. Perceived organizational structure was associated with greater workplace efficacy, and this relationship was equally strong for veterans and civilians, see Fig 1a.

#### Perceived structure predicts sense of belonging.

Next, we examined whether perceived organizational structure was associated with belonging at the workplace, and whether this relationship varied as a function of veteran status. We conducted a second hierarchical linear regression to examine whether veteran status moderated the association between perceived organizational structure and sense of belonging. We entered veteran status (1 =  veteran and 0 =  civilian) and mean-centered perceived organizational structure as predictors at Step 1 and their interaction as an additional predictor at Step 2. Sense of belonging was entered as the outcome variable. From Step 1, there was a significant main effect of perceived organizational structure, *β* =  0.64, *b* =  0.78, *SE* =  0.06, *t*(242) =  13.02, *p* <  0.001, 95% CI for *b* =  [0.66, 0.90]. Participants who perceived greater structure at their first civilian organization reported feeling a greater sense of belonging in that workplace. The main effect of veteran status was not significant, *β* =  -0.05, *b* =  -0.12, *SE* =  0.13, *t*(242) =  0.93, *p* =  0.36, 95% CI for *b* =  [-0.39, 0.14].

Importantly, from Step 2, the main effect of perceived organizational structure was qualified by a significant interaction between perceived organizational structure and veteran status, *β* =  -0.13, *b* =  -0.27, *SE* =  0.13, *t*(241) =  2.13, *p* =  0.03, 95% CI for *b* =  [-0.52, -0.02]. Perceived organizational structure was more strongly associated with sense of belonging among veterans, *b* =  0.87, *SE* =  0.07, *t*(241) =  11.92, *p* <  0.001, compared to civilians, *b* =  0.60, *SE* =  0.10, *t*(241) =  5.80, *p* <  0.001. While perceiving organizational structure in one’s first place of employment was associated with a greater feeling of belonging among all participants, this relationship was significantly stronger for military veterans compared to civilians, see Fig 1b.

#### Occupational self-efficacy mediates perceived structure and sense of belonging relationship.

**Fig 2:**
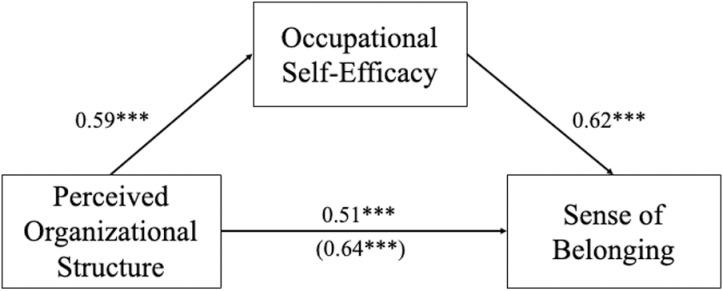
The effect of structure on belonging, mediated through efficacy. Path coefficients are standardized regression coefficients. The total effect relating perceived organizational structure to belonging is shown in parentheses. The model also included age, gender, and race as covariates. ***p <  0.001.

Finally, we tested a mediational model where perceived organizational structure predicted sense of belonging, mediated through occupational self-efficacy using ordinary least squared regression. We first regressed sense of belonging on perceived organizational structure (mean-centered). There was a significant main effect of perceived structure, *β* =  0.64, *b* =  0.78, *SE* =  0.06, *t*(243) =  13.01, *p* <  0.001, 95% CI for *b* =  [0.66, 0.90]. Second, we regressed occupational self-efficacy on perceived organizational structure (mean-centered). Once again there was a significant main effect of perceived structure, *β* =  0.59, *b* =  0.56, *SE* =  0.05, *t*(243) =  11.53, *p* <  0.001, 95% CI for *b* =  [0.46, 0.65]. Finally, we regressed sense of belonging on occupational self-efficacy. There was a significant main effect of self-efficacy, *β* =  0.62, *b* =  0.68, *SE* =  0.07, *t*(244) =  9.48, *p* <  0.001, 95% CI for *b* =  [0.54, 0.82]. A bootstrap confidence interval (based on 5,000 samples) for the standardized indirect effect, *β* =  0.31, *SE* =  0.08, did not include zero, 95% CI for *β* =  [0.16, 0.46], providing evidence consistent with the proposed mediation model. Perceived organizational structure was associated with sense of belonging partially as a result of its relationship with occupational self-efficacy. However, even after controlling for occupational self-efficacy, there remained a significant (though reduced) direct association between perceived organizational structure and belonging, *β* =  0.51, *b* =  0.63, *SE* =  0.07, *t*(242) =  8.60, *p* <  0.001, 95% CI for *b* =  [0.48, 0.77]. See Fig 2 for a visual depiction of the model. This mediational relationship was consistent for both veterans (*β*_indirect effect_ =  0.31, *SE* =  0.10, 95% CI for *β* =  [0.12, 0.50]) and civilians, *β*_indirect effect_ =  0.31, *SE* =  0.12, 95% CI for *β* =  [0.10, 0.59].

This analysis provides support for the hypothesized mediational relationship between perceived structure, efficacy, and belonging. Occupational self-efficacy appears to partially mediate the relationship between perceived organizational structure and sense of belonging such that structure is associated with greater efficacy, which in turn predicts greater belonging. The relationship between organizational structure and occupational efficacy was consistent for veterans and civilians, and so there was no moderation of the mediation. Transitioning to an environment that provides greater structure will be associated with more positive psychological outcomes, specifically greater self-efficacy and, in turn, greater belonging.

#### Additional analyses.

Study 1 identified a positive relationship between organizational structure, occupational efficacy, and belonging. People who perceived greater organizational structure also felt a greater sense of belonging at those first organizations as they were transitioning, and they felt more efficacious in their jobs, whereas people who saw their organization as less structured, felt less efficacious and belonging. Study 1 also provides initial evidence that the relationship between organizational structure and sense of belonging may be particularly strong for veterans in their career transition, as reflected in the veteran status by structure interaction in predicting belonging. One possibility for this relationship was suggested in additional analyses with a subset of participants (*N* =  180; 148 veterans, 32 civilians; the low number of civilians who received this measure was due to a coding error in the creation of the survey that was not initially recognized) who indicated their perceived structure in both the military (for veterans) and their education (for civilians). Veterans decreased in perceived structure from the military to their first civilian job (*p* =  0.03) whereas civilian students non-significantly increased in their perceived structure from their education to their first post-graduation job (*p* =  0.29), resulting in a marginally significant veteran status by context interaction, *F*(1, 178) =  3.27, *p* =  0.07, *η*^2^ =  0.01 (see Study 1 Supplemental Analysis in supplemental materials for full results). One possibility, then, is that a structured environment may be particularly effective at reminding veterans of a sense of a community they experienced as part of their previous military experience, promoting stronger person-environment fit [67]. This similarity to one’s previous work environment may have, in turn, supported a feeling of belonging in veterans’ new civilian work.

Study 1 provides some preliminary support for the notion that individuals who have been socialized to expect significant structure in their work environment (i.e., military veterans) will respond more positively to structured civilian work environments as perceived organizational structure was more strongly associated with sense of belonging among veterans compared to civilians. It is important to note, however, that there is a great deal of heterogeneity among military veterans that may contribute to differences in the impact of structure on workplace outcomes. In an effort to investigate how heterogeneity among veterans and their military experience may influence how post-military employment structure predicts efficacy and belonging, we conducted an additional correlational study with a sample of 340 veterans (which we report in supplemental materials; Supplemental Study 1). Recruiting a sample of veterans with significant variability in military experience, including branch and era of service, as well as service role (e.g., Administrative, Support, Logistics; Combat Operations, Infantry, Pilot), we found consistent evidence that perceived organizational structure predicted greater occupational self-efficacy which, in turn, predicted greater sense of belonging. This suggests that regardless of the nature of a veterans’ military experience, their perception of their own work performance in a new civilian position and their sense of belonging in their new workplace is associated with the amount of structure they perceive in their new work environment. A full description of this study and findings is included in supplemental materials (see Supplemental Study 1).

## Study 2

In Study 2, we utilized a unique data collection opportunity to examine the relationship between perceived structure, efficacy, and belonging at veterans’ current (as opposed to previous) employers. In Study 2 (preregistration available on OSF), we explore whether military veterans’ occupational efficacy and sense of belonging may continue to be associated with their perceptions of organizational structure well past their initial transition. As such, we explore if perceived organizational structure would be positively associated with occupational efficacy and, in turn, sense of belonging, among a sample of veterans considering their current organization. In sum, this methodological change, to focus on veterans’ current job could assuage some concerns about biases in memory that may have occurred in Study 1. Study 2 enabled us to replicate the findings of the previous studies with an additional large sample, pre-register our analytic plan, and examine the robustness of the mediational relationship observed in Study 1 (and Supplemental Study 1).

### Methods

#### Participants.

A sample of 497 U.S. military veterans were recruited by VetsinTech and Center for a New American Security (CNAS; see Table 1 for sample demographics). VetsinTech provides re-integration services to current and returning veterans, specializing in connecting veterans to opportunities in the technology sector. CNAS is an independent, bipartisan, nonprofit think tank that develops national security and defense policies. Data collection began on August 26^th^, 2021, and concluded on October 3rd, 2021. No data analyses were conducted prior to the completion of data collection. This sample size of 497 participants provided 80% power for detecting an effect (*r*) as small as 0.13. Participation was on a voluntary (i.e., no compensation) basis among people invited to participate by VetsinTech. In addition, we assessed personal need for structure, and that was described in the pre-reg as a moderator. That is not the focus of the current manuscript and is not discussed further. The mediation is pre-registered (see OSF).

#### Measures.

Consenting participants completed an online survey examining “technology-related skills gained during their time in service and their perceptions of a future career or experience in the technology sector.”

Because of the nature of data collection, participants responded to single-item measures of the key constructs of perceived organizational structure, occupational self-efficacy, and sense of belonging in their current civilian work. Participants’ perception of structure at their current organization was measured by the item “My current organization provides a clear and structured everyday life” [45]. Participants’ sense of being efficacious at their current workplace was measured by the item “Whatever comes my way in my job, I can usually handle it” [42]. Finally, participants’ sense of belonging at their current organization was measured by the item “I feel like I belong at my current organization” [66]. We chose these items based on their face validity and examining the findings from Study 1. All three items were measured on seven-point scales ranging from 1 (*strongly disagree*) to 7 (*strongly agree*).

### Results

We tested the proposed mediational model where perceived organizational structure at veterans’ current employer predicted sense of belonging, mediated through occupational self-efficacy. Ordinary least squared regression was used. We first regressed sense of belonging on perceived organizational structure (mean-centered) controlling for age (0 =  over 50 years old, 1 =  under 50 years old), gender (0 =  male, 1 =  non-male), and race (0 =  White/Caucasian, 1 =  non- White/Caucasian). The racial categories used in Study 2 differ from Studies 1 & 3 because they were chosen by the organization that fielded the survey. There was a significant main effect of perceived structure, *β* =  0.37, *b* =  0.38, *SE* =  0.04, *t*(492) =  8.93, *p* <  0.001, 95% CI for *b* =  [0.29, 0.46]. Participants who perceived greater structure in their current employer reported a greater sense of belonging in their workplace. Second, we regressed occupational self-efficacy on perceived organizational structure (mean-centered; with the same covariates as in the previous analysis). There was a marginally significant main effect of perceived structure, *β* =  0.08, *b* =  0.04, *SE* =  0.02, *t*(492) =  1.84, *p* =  0.07, 95% CI for *b* =  [-0.003, 0.09]. Participants who perceived greater structure in their current employer reported feeling somewhat greater efficacy in their work. Finally, we regressed sense of belonging on occupational self-efficacy (with the same covariates as in the previous analyses). There was a significant main effect of self-efficacy, *β* =  0.29, *b* =  0.54, *SE* =  0.08, *t*(492) =  6.51, *p* <  0.001, 95% CI for *b* =  [0.38, 0.70].

**Fig 3:**
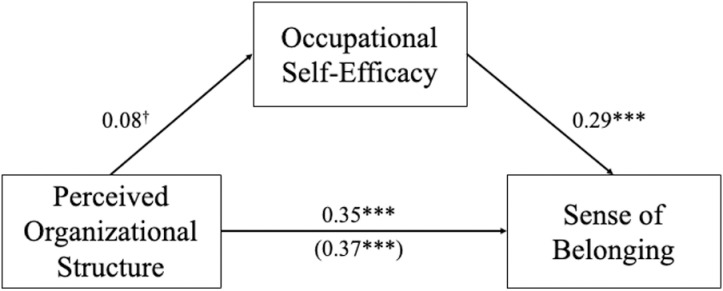
The effect of structure on belonging, mediated through efficacy. Path coefficients are standardized regression coefficients. The total effect relating perceived organizational structure to belonging is shown in parentheses. The model also included age, gender, and race as covariates. ***p <  0.001, ^†^0.05 *<  p* <  0.10.

A bootstrap confidence interval (based on 5,000 samples) for the standardized indirect effect, *β* =  0.02, *SE* =  0.01, did not include zero, 95% CI for *β* =  [0.001, 0.06], providing evidence consistent with the proposed mediation model. Perceived organizational structure is associated with sense of belonging partially as a result of its relationship with occupational self-efficacy. However, even after controlling for occupational self-efficacy, there remained a significant (though reduced) direct association between perceived organizational structure and belonging, *β* =  0.35, *b* =  0.35, *SE* =  0.04, *t*(491) =  8.72, *p* <  0.001, 95% CI for *b* =  [0.27, 0.43]. See Fig 3 for a visual depiction of the model.

Predictions regarding the moderating role of personal need for structure were preregistered for Studies 2 and 3. These predictions are not investigated here as it is not the focus of the present manuscript. Additionally, there is a discrepancy between the sample size reported in Study 2’s preregistration and that reported in the present analyses. The sample size reported in the preregistration was incorrectly calculated as it included survey participants who were both military veterans and those actively serving in the military and/or reserves. The analyses reported here only used data from participants who were fully separated from the military.

Study 2 demonstrated positive associations between veterans’ perceived structure in their current civilian organization and sense of belonging as well as occupational efficacy. Study 2 additionally provides evidence consistent with the hypothesized mediational relationship, finding that perceived structure at veterans’ current organization is associated with greater occupational self-efficacy, which in turn promotes a greater sense of belonging. These results suggest that providing a structured work environment to veterans, who have been socialized to expect continued structure in their employment, past their initial transition to a civilian employer continues to be associated with certain beneficial outcomes. Likewise, if structure does not continue to be provided to veterans in their civilian work, this is associated with more detrimental outcomes (reduced belonging and efficacy). Of course, reverse causality, that those who are feeling more efficacy and belonging perceive greater structure in their workplaces cannot be accounted for with the correlational design. Thus, we sought to examine whether there is a causal relationship between perceived structure and these variables in Study 3.

## Study 3

Study 3 investigates how manipulated perceived organizational structure relates to occupational self-efficacy and belonging, going beyond Studies 1 and 2 that are both correlational and either retrospective (Studies 1) or cross-sectional (Study 2) in design. Study 3 (preregistration available on OSF) investigates the causal relationship between perceived structure and efficacy and belonging. We hypothesized that participants would report greater anticipated occupational self-efficacy and sense of belonging in the job when it emphasizes greater structure. Likewise, following the initial evidence that structure may be particularly beneficial among veterans on belonging in the organization, relative to civilians, found in Study 1, we also hypothesized and tested whether emphasizing structure (vs. not emphasizing structure) would be more beneficial to efficacy and belonging for military veterans than civilians.

### Methods

#### Participants.

An international sample of 200 military veterans were recruited from Prolific (172) and through snowball sampling on LinkedIn (28) and 200 civilians were recruited using Prolific (see Table 1 for sample demographics and nationality information). Data collection began on March 1st, 2023, and concluded on April 26th, 2023. We turned to LinkedIn for recruiting veterans when it became difficult to obtain the desired sample size of veterans from Prolific. No data analyses were conducted prior to the completion of data collection. Our final sample size provided 80% power for detecting an effect (*r*) as small as 0.14. Participants recruited through Prolific were compensated between $3.25 and $5.00 for their participation while participants recruited through snowball sampling were not compensated, that is, they participated as volunteers.

#### Measures.

Consenting participants completed an online survey examining “factors that may influence how people view different job opportunities and experiences.” To keep the survey concrete and engaging, and to remind participants what organization they were evaluating, participants provided the initials of their current civilian employer. Likewise, civilian participants were asked to provide the initials of the school/university they most recently attended. These initials were used to personalize the wording of some items.

Following this, participants were given a series of writing prompts where they were asked to reflect on their previous experiences in the military or in the education system. Veterans responded to “Please describe your job in the military” and “Please describe your favorite memory of being in the military.” These questions were intended to remind veteran participants of their veteran status. Civilians responded to “Please describe your academic activities at [YY; Participant Provided School/University Initials]” and “Please describe your favorite memory of being at YY.”

Next, participants were randomly assigned to either a high or low structure condition. The manipulation created conditions where participants would believe they would have high (or low) efficacy in a new position. All participants imagined they were starting a new job at “Company GC,” a fictitious company. Participants then read a welcome email from their new manager, see Table 3.

**Table 3:** Study 3 structure condition messages.

High structure condition	Low structure condition
Hi, Welcome to Company GC. I hope you’re settling in well. We recently signed a contract with a major new client. You were hired to act as the point of contact for this new client. *You will be expected to facilitate communication between the client and GC, answer questions the client may have, and help to ensure that we are meeting the client’s expectations*. This is in keeping with the *service-delivery* nature of our company. I will be introducing you to the client at a meeting tomorrow. Please put together one presentation slide about your role that I can include in our slide deck for the meeting. Thanks, Management, GC	Hi, Welcome to Company GC. I hope you’re settling in well. We recently signed a contract with a major new client. You were hired to act as the point of contact for this new client. *As this is a new client, we are not entirely certain what you can expect this job to entail*. *There are no specific expectations for your role at this point*. This is in keeping with the *free-flowing* nature of our company. I will be introducing you to the client at a meeting tomorrow. Please put together one presentation slide about your role that I can include in our slide deck for the meeting. Thanks, Management, GC

Messages from fictitious managers in the high and low self-efficacy conditions. Differences between conditions are italicized.

Next, participants reported their anticipated sense of efficacy in the job using three-items adapted from the short form Occupational Self-Efficacy Scale [42]. The three items were: “I can handle whatever comes my way in this job at Company GC,” “I would be able to remain calm when facing difficulties in this job at Company GC because I can rely on my abilities,” and “I feel prepared for my occupational future at Company GC.” All items were measured on seven-point scales ranging from 1 (*strongly disagree*) to 7 (*strongly agree*). The scores of the three items were averaged to generate a composite, *M* =  5.19, *SD* =  1.25, α =  0.87.

Then, participants reported their anticipated sense of belonging at the fictitious company using a three-items adapted from the Sense of Social and Academic Fit scale [66]. The three items were “I would feel like I belong at Company GC,” “I would fit in well at Company GC,” and “I would feel comfortable at Company GC.” All three items were measured on seven-point scales ranging from 1 (*strongly disagree*) to 7 (*strongly agree*). The scores of the three items were averaged to generate a composite, *M* =  4.63, *SD* =  1.29, α =  0.93.

Finally, participants completed a single item manipulation check, “How much structure do you think there will be in your new job at Company GC?” measured on a seven-point scale ranging from 1 (*none at all*) to 7 (*a great deal*).

### Results

#### Manipulation check.

An independent samples t-test was used to test for condition differences on the manipulation check item. As expected, participants in the high structure condition anticipated greater structure in the described job (*M* =  4.88, *SD* =  1.19) compared to those in the low structure condition (*M* =  3.26, *SD* =  1.31), *t*(395) =  12.89, *p* <  0.001, *d* =  1.29.

#### Condition effects on efficacy.

To test if anticipating working at a more (vs. less) structured job leads to greater anticipated efficacy and belonging at that job, and if this relationship is stronger for military veterans, two two-way ANOVAs were conducted. We report ANOVA results as the primary analyses and include ANCOVA results in supplemental materials (note the ANCOVA analyses were pre-registered, however, the results are robust to the inclusion of covariates). Experimental condition and veteran status were used to predict anticipated occupational self-efficacy and, separately, sense of belonging. Results revealed significant main effects of both condition, *F*(1, 396) =  24.13, *p* <  0.001, η^2^ =  0.051, and veteran status, *F*(1, 396) =  40.62, *p* <  0.001, η^2^ =  0.087 on anticipated efficacy. Participants in the high structure condition anticipated greater efficacy at the fictitious company (*M* =  5.48, *SD* =  1.08) compared to those in the low structure condition (*M* =  4.91, *SD* =  1.33), while veteran participants overall anticipated greater efficacy at the fictitious company (*M* =  5.56, *SD* =  1.07) compared to civilian participants (*M* =  4.82, *SD* =  1.30). The interaction between condition and veteran status was marginally significant, *F*(1, 396) =  3.74, *p* =  0.054, η^2^ =  0.008. Tukey’s HSD post-hoc tests showed that compared to the low structure condition, the high structure condition significantly increased non-veterans’ anticipated efficacy, *p* <  0.001, but did not significantly increased veterans’ anticipated efficacy, *p* =  0.160. However, directionally, and consistent with the main effect results, both groups reported greater self-efficacy in the high structure condition than the low structure condition. The lack of a significant effect among veterans may be explained by veterans’ overall higher levels of self-efficacy, regardless of condition.

#### Condition effects on belonging.

Results of the second ANOVA revealed a significant main effect of condition, *F*(1, 396) =  19.30, *p* <  0.001, η^2^ =  0.046, and a marginally significant main effect of veteran status, *F*(1, 396) =  3.43, *p* =  0.065, η^2^ =  0.008, on anticipated belonging. Participants in the high structure condition anticipated greater belonging at the fictitious company (*M* =  4.90, *SD* =  1.10) compared to those in the low structure condition (*M* =  4.35, *SD* =  1.40), while veteran participants overall anticipated marginally greater belonging at the fictitious company (*M* =  4.74, *SD* =  1.22) compared to civilian participants (*M* =  4.51, *SD* =  1.34). The interaction between condition and veteran status was non-significant, *F*(1, 396) =  0.44, *p* =  0.506, η^2^ =  0.001.

#### Occupational self-efficacy mediates manipulated structure and sense of belonging relationship.

**Fig 4:**
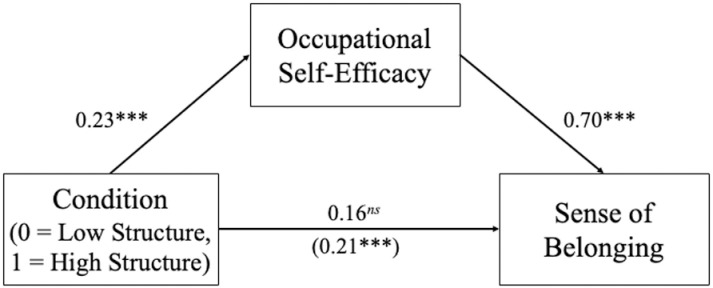
The effect of manipulated structure on anticipated belonging, mediated through efficacy. Path coefficients are standardized regression coefficients. The total effect relating perceived organizational structure to belonging is shown in parentheses. ***p <  0.001, ^*ns*^*p* >  0.10.

Finally, we tested an exploratory (i.e., not preregistered) mediational model where condition predicted sense of belonging, mediated through occupational self-efficacy using ordinary least squared regression. We first regressed sense of belonging on condition (0 =  Low Structure, 1 =  High Structure). There was a significant main effect of condition, *β* =  0.21, *b* =  0.55, *SE* =  0.13, *t*(398) =  4.38, *p* <  0.001, 95% CI for *b* =  [0.30, 0.80]. Second, we regressed occupational self-efficacy on condition. Once again there was a significant main effect of condition, *β* =  0.23, *b* =  0.57, *SE* =  0.12, *t*(398) =  4.67, *p* <  0.001, 95% CI for *b* =  [0.33, 0.81]. Finally, we regressed sense of belonging on occupational self-efficacy. There was a significant main effect of self-efficacy, *β* =  0.70, *b* =  0.72, *SE* =  0.04, *t*(398) =  19.48, *p* <  0.001, 95% CI for *b* =  [0.65, 0.79]. A bootstrap confidence interval (based on 5,000 samples) for the standardized indirect effect, *β* =  0.16, *SE* =  0.03, did not include zero, 95% CI for *β* =  [0.09, 0.22], providing evidence consistent with the proposed mediation model. Condition was associated with sense of belonging as a result of its relationship with occupational self-efficacy. After controlling for occupational self-efficacy, there remained no significant direct association between condition and belonging, *β* =  0.06, *b* =  0.15, *SE* =  0.09, *t*(397) =  1.59, *p* =  0.113, 95% CI for *b* =  [-0.04, 0.34]. See Fig 4 for a visual depiction of the model. This mediational relationship was consistent for both veterans (*β*_indirect effect_ =  0.10, *SE* =  0.04, 95% CI for *β* =  [0.01, 0.18]) and civilians, *β*_indirect effect_ =  0.24, *SE* =  0.05, 95% CI for *β* =  [0.13, 0.33].

#### Additional analyses.

Supporting our theory and predictions, participants in the high structure condition reported significantly greater anticipated efficacy and belonging in the hypothetical job compared to those in the low structure condition. However, counter to our hypothesis, we also observed that the high structure condition seemed to be slightly more impactful for increasing civilians’ anticipated efficacy compared to veterans. One possibility is that this may have been the result of a ceiling effect among the veteran sample. That is, there was a significant main effect of veteran status on both efficacy and belonging such that veterans, collapsing across experimental condition, reported greater anticipated efficacy and belonging compared to civilians. Thus, the effect of the experimental condition may have been constrained by the veterans’ overall higher levels of efficacy.

Why might veterans have had higher overall anticipated efficacy and belonging than civilians? One possible explanation may be linked to the amount of time the veterans in our sample have spent out of military service. The Study 3 sample of veterans spent, on average, 12.8 years (*SD* =  11.0) out of service. As such, it’s likely these veterans have gone through at least one, if not several, career transitions, though it is impossible to know this conclusively as we did not ask participants about past employment changes. This possible experience of having gone through previous transitions may have taught the veterans in our sample that despite the challenges they may face, they will eventually be successful. This may have resulted in the veterans reporting higher anticipated efficacy and belonging overall, because of their previous experience and success with employment transitions. Despite this higher average level of efficacy and belonging compared to the civilians sampled, and despite the considerable average time the veterans we sampled had spent out of military service, being in the high structure condition resulted in significantly greater anticipated belonging for the veteran participants in Study 3 relative to those in the low structure condition. A structured work environment provides benefits to veterans who face an employment transition even years after their military discharge. Study 3 thus provides evidence of perceived employment structure causing anticipated efficacy and belonging during a career transition.

## General discussion

Across three studies (plus one supplemental study) we investigated how the structure within an organization, as perceived by the employee, could help people compensate for the uncertainty involved in employment transitions. We examined whether greater perceptions of structure, whether measured or manipulated, are associated with improved transition outcomes in the form of occupational self-efficacy and sense of belonging, especially among military veterans who have been theorized to experience greater loss of employment structure during employment transition [3,24]. Using correlational designs, Studies 1 and 2 provide evidence that perceiving greater structure at one’s organization as an employee is associated with greater self-efficacy which, in turn, is associated with greater belonging. Additionally, using an experimental design, Study 3 provides evidence that perceiving greater structure in one’s new work environment facilitates increased feelings of occupational self-efficacy and sense of belonging. The three studies employed both correlational and experimental methodologies to assess empirically the relationships between the predictor variable (perceived structure), the mediating variable (efficacy at work), and the outcome variable (belonging at work). Building on foundational ideas in compensatory control theory [8,32] as to how people cope with a perceived loss of control by finding other sources of control in their environment, this research makes a number of applied and theoretical contributions and raise important questions for future research.

### Implications for compensatory control theory

The present studies contribute to compensatory control theory in three ways. First, belonging is a central motivating factor in people’s lives [46,47], and yet, it has not been considered in relation to models of compensatory control, which are built on the fundamental motive to perceive the world as orderly [9–12]. Building on prior theorizing that applies compensatory control theory [8] to the experience of the workplace [45], the research here provides evidence that variables that help people compensate for losses in personal control (perceived structure) are related (and in Study 3, causally) to belonging. Given the centrality of belonging to performance and well-being [66,68,69] this is an important theoretical development.

Second, CCT research has only recently begun to explore how one’s cultural environment shapes control compensation behavior [70]. For example, Ma et al. [71] investigated how tight vs loose cultural contexts shape control compensation processes. By focusing on veterans, who have experience in tight cultures (i.e., the military) [72], the present work expands understanding of how a structured environment may compensate for low control to influence important outcomes.

Finally, CCT research has broadly focused on the phenomena of low control while theorizing about the implications of specific real-world manifestations of low control. The compensatory control process has rarely been investigated in the context of a specific form of lost control [73]. The present research does this by focusing on life transitions, specifically the loss of control that may occur during employment transitions.

### Evidence for differential effects among veterans and civilians

The present work treated military veterans as the primary group of interest. As previously discussed, we theorized that the total institution of the military socializes service members to expect a high degree of structure from their employment environment, due to the regimented, structured, and hierarchical nature of the military [16–20]. Recognizing that most civilian employers would be unable to match the level of structure provided by the military [24], implying a loss of environmental structure when service members transition to being civilian employed veterans, we hypothesized that veterans would particularly benefit from a highly structured civilian work environment, relative to their civilian counterparts who were not socialized to expect structure in their employment environment. While Study 1 provided some evidence that structure may be more strongly associated with sense of belonging at work among veterans compared to civilians, Study 3 failed to find causal evidence for structure differentially promoting veterans’ and civilians’ anticipated occupational self-efficacy and sense of belonging. Furthermore, in Study 3 veterans reported higher overall levels of anticipated efficacy and belonging compared to civilians.

Taken together, the present research provides evidence that structure appears to be beneficial for both veterans and civilians and does not provide strong evidence that structure is particularly beneficial in promoting employment outcomes among military veterans. However, there are some limitations of the present samples that may explain this lack of differential effects. Most importantly, most veterans sampled had been separated from the military for several years, and in many cases for over a decade (see Table 2). This considerable time away from the military may have led to a weakening of the socialization veterans experienced while they were serving. It’s possible that because of this lengthy separation and possible socialization weakening, veterans, despite their unique employment history, were influenced by employment structure similarly to non-military civilians. Sampling newly separated veterans, among whom a stronger benefit of a structured work environment would be predicted, and following them longitudinally to determine their (non-retrospective) work outcomes is an important avenue for future research.

Another possibility to consider in terms of the generally null findings between veterans and non-veterans is the appropriate comparison group. In the present studies, we focused (in Studies 1 and 3) on people who reflected on their transition from college to work settings as the comparison group. While it seems plausible that one of the biggest factors distinguishing the college transition from the military transition is the relative amount of structure in each environment, that assumption too would be better tested by contemporaneous assessments of perceived structure among the transitioning groups and longitudinal assessments. In addition, having multiple comparison groups (e.g., people retiring from a professional sports team) would enable stronger inferences.

Thus, the initial inference from the present research is that there are benefits on belonging through increased efficacy of perceiving one’s new work environment as being structured for both veteran and civilian employees. To the extent employers can help employees perceive greater structure in their work, possibly by leveraging *actual* organizational structure, this may promote improved employment outcomes among employees of varying backgrounds. Future research may provide stronger tests of whether this is particularly important for people transitioning from high structure environments, such as the military.

### Open questions and future directions

In addition to the issues outlined above, there are several other applied and theoretical research questions and future directions we would like to highlight.

First, while these studies demonstrate the importance of general perceived structure in facilitating positive employment transition outcomes, they do not provide insight into the specific forms of structure that best facilitate these outcomes. The measure of structure used in the current studies combines multiple facets of organizational structure including hierarchy, role responsibilities/routine, and organization policies/rules [45,74]. Each of these specific forms of structure may contribute to the development of improved transition outcomes in disparate ways. For example, while specification of role responsibilities may be particularly beneficial for developing occupational self-efficacy, clarity surrounding organizational culture and policies may be more beneficial for the development of one’s sense of belonging. Likewise, recent theoretical work on CCT has suggested that depending on an individual’s cultural worldview, different compensatory responses to low control may be particularly beneficial [70]. As such, it may be the case that the specific form of organizational structure that is most beneficial during employment transitions may vary between populations (e.g., between veterans and civilians). Future research would benefit from investigating the nuanced influence of specific forms of organizational structure on employment outcomes.

Second, the present studies focused specifically on employees’ *perception* of structure at their past (Study 1), present (Study 2), and hypothetical (Study 3) organizations. It was found that manipulation of perceived structure was sufficient to influence anticipated occupational efficacy and belonging. While the present work focused on perceptions of structure, there is an important extant literature on how *actual* organizational structure can impact processes of information processing, decision making, and employee performance [33–37]. The findings of the present work raise the question of the relationship between actual and perceived structure. While in general, one would assume that changes in actual structure are reflected in changes in perceived structure, there may be variability among people and situations in the extent to which this is the case. One could imagine situations where changes in actual structure are clearly announced by an organization and result in changes in perceived structure – as well as situations when actual structural changes are more “behind the scenes.” We would suggest that to the extent changes in actual organizational structure are identified and perceived by employees, this may partially explain the mechanism by which actual employment structure influences employee outcomes, but this is an open and important question. As Brockner and Sherman [48] outline in their review of wise interventions in organizations, they can occur at both the structural level as well as at the level of individual construal of the situation. Researchers and practitioners seeking to apply the present work should consider how these two pathways-to-change influence transitioning employees.

Third, throughout each of the studies presented, a strong relationship between occupational self-efficacy and sense of belonging was observed. The present work suggests that interventions designed to strengthen organizational structure during employment transitions could be paired with interventions designed to secure feelings of belonging [66,68] and efficacy to mutually improve workplace efficacy and belonging. The integration of organizational interventions is an exciting area for future research [48].

Finally, while the samples used in the present studies provided unique research opportunities, they also present important limitations to generalizability and inference. Each sample provided a reasonably diverse sample of military veterans and across studies, questions addressed veterans’ past, present, and hypothetical future careers. Observing evidence of the theorized relationship between structure, efficacy, and belonging across each of these contexts among diverse samples provides convergent validity for the proposed model. However, while providing convergent evidence for the proposed hypotheses, heterogeneous effects among important subgroups were left unexplored outside of Supplemental Study 1 (which showed convergent results across branch and era of service, service role, and if a veteran joined the reserves immediately after service). Future research is needed to ensure the results observed in the present studies generalize appropriately to all groups of veterans, particularly recently discharged veterans as noted above. Additionally, our theoretical predictions focus on the employment transition window, and none of the studies presented included longitudinal data collection during employment transition. To understand the processes at play during employment transitions more acutely, robust longitudinal studies, measuring both perceived and actual employment structure as well as sampling both transitioning veterans and civilians, would enable greater generalizability and more precise theory testing. Longitudinal data from both veterans and civilians experiencing employment transition would allow for a better understanding of the challenges both groups face, what challenges are unique to each group, and how structure may play a role in facilitating transition outcomes over time.

## Conclusion

Transitioning to a new job presents significant challenges, especially for individuals who have recently concluded their time serving their country in the military [3,7]. Yet, the present research suggests a unique opportunity for employers to consider through the promotion of structure to not only help transitioning employees face the challenges their transitions present, but to also promote greater success in their new jobs. This could be achieved during onboarding processes that communicate the core elements of the organization’s structure that might not be apparent early in employment. The research presented in this paper supports onboarding approaches that carefully consider such factors. Across three studies with varying methodologies, we have provided evidence of the important role that organizational structure can play in facilitating occupational self-efficacy and sense of belonging among transitioning employees, focusing on the perceptions of military veterans. As organizations seek to improve the transition outcomes of new hires, and to benefit from the unique abilities and skillsets veterans possess, the research presented here argues for careful consideration of the structure organizations provide their employees and the consequences this may bring.
